# Stress-specific p38 MAPK activation is sufficient to drive EGFR endocytosis but not its nuclear translocation

**DOI:** 10.1242/jcs.202358

**Published:** 2017-08-01

**Authors:** Alejandra Tomas, Sylwia Jones, Simon O. Vaughan, Daniel Hochhauser, Clare E. Futter

**Affiliations:** 1 UCL Institute of Ophthalmology, University College London, 11-43 Bath Street, London EC1V 9EL, UK; 2 Cancer Research UK Drug-DNA Interactions Research Group, UCL Cancer Institute, Paul O'Gorman Building, University College London, 72 Huntley Street, London WC1E 6BT, UK

**Keywords:** EGF receptor, p38 MAPK, Endocytosis, Nuclear translocation

## Abstract

EGF receptor (EGFR) endocytosis is induced by stress in a manner dependent on the p38 MAPK family. Ligand and stresses such as X-rays, reportedly promote nuclear trafficking of endocytosed EGFR for regulation of gene transcription and DNA repair. We fail to detect EGFR endocytosis or nuclear transport following X-ray treatment of HeLa or head and neck cancer cells, despite extensive DNA damage induction. Apparent nuclear staining with EGFR extracellular domain antibody remained present despite reduced/absent EGFR expression, and so did not represent nuclear EGFR. UVB and UVC, but not X-ray or UVA, treatment induced p38 activation and EGFR endocytosis, although all of these stresses induced DNA damage, indicating that DNA damage alone is not sufficient to induce EGFR endocytosis. Increased reactive oxygen species (ROS) levels following UVB treatment, compared to that seen with X-rays, do not alone explain differences in p38 activation. UVB, like UVC, induced EGFR accumulation predominantly in perinuclear endosomes, rather than in the nucleus. Our morphological techniques identifying major changes in receptor distribution do not exclude the possibility that small but biologically relevant amounts of EGFR enter the nucleus. This study highlights the importance and limitations of morphological analyses of receptor distribution in understanding signaling outcome.

## Abstract (precis)

**Highlighted Article:** The DNA damage-inducing agents UVB and UVC, but not X-rays or UVA, activate p38 MAPK and drive EGFR endocytosis without detectable EGFR nuclear translocation. Hence, DNA damage alone is not sufficient to induce EGFR endocytosis.

## INTRODUCTION

The ligand-stimulated endocytosis of EGF receptor (EGFR) and endosomal sorting complexes required for transport (ESCRT)-dependent delivery to lysosomes is a well-described pathway that is known to regulate EGFR signaling (Tomas et al., 2014). EGFR endocytosis can also be promoted by various stresses that induce DNA damage, including ultraviolet (UV) light, X-rays and chemotherapeutic agents like cisplatin. The fates of endocytosed EGFR induced by stress are less well-characterized, but have been reported to include import into the nucleus where the receptor can promote transcription of genes associated with cell proliferation and also promote DNA repair (Wang and Hung, 2012). The transport of full-length EGFR to the nucleus remains controversial despite being widely reported following exposure not only to DNA damage-inducing agents (Dittmann et al., 2008; Hsu et al., 2009; Liccardi et al., 2011; Xu et al., 2009) but also to ligand (Liao and Carpenter, 2007; Lin et al., 2001). Although EGFR trafficking in response to stress is a topic of comparatively recent interest, the trafficking of ligand-stimulated receptor has been extensively studied over many years and in the majority of these studies nuclear trafficking was not reported. One possible reason for this discrepancy is that, according to some reports, only a small proportion of EGFR traffics to the nucleus and this can take many hours (Liao and Carpenter, 2007), whereas most analyses of EGFR endocytosis have focused on events occurring within the first 1–2 h. However, other studies report transport of EGFR to the nucleus within minutes of EGF stimulation (Lin et al., 2001).

Nuclear EGFR is of huge potential interest in the diagnosis and treatment of cancer. EGFR is overexpressed in ∼50% of human cancers and is a target for cancer therapies. Many studies have reported nuclear EGFR in tumors where it is associated with poor prognosis (Lo et al., 2005; Xia et al., 2009). Nuclear transport of substantial amounts of EGFR after treatment of cultured cells with X-rays or the chemotherapeutic drug cisplatin, has been implicated in the repair of double-stranded DNA breaks through interaction with DNA-dependent protein kinase (DNA-PK) and PCNA (Hsu et al., 2009; Liccardi et al., 2011; Wang et al., 2012). This provides a potential mechanism whereby anti-EGFR therapeutics that inhibit EGFR trafficking and/or signaling could potentiate the effects of X-rays and chemotherapeutics aimed at inducing tumor cell death through DNA damage.

One reason for skepticism concerning the possibility of full-length EGFR entering the nucleus has been the conundrum of how the receptor can be extracted from the membrane, enter the nucleus and accumulate in the nucleoplasm. Evidence for a potential pathway has accumulated over the last 10 years. Although the initial step of endocytosis has been investigated for both ligand-stimulated and stress-induced EGFR, the majority of studies of the post-endocytic traffic of EGFR to the nucleus have focused on EGF-stimulated EGFR. Following endocytosis, either by clathrin-coated pits or caveolae, EGFR is proposed to undergo syntaxin 6-dependent trafficking from early endosomes to the Golgi (Du et al., 2014), followed by COP1-dependent retrograde trafficking to the endoplasmic reticulum (ER) (Liao and Carpenter, 2007; Wang et al., 2010a). This is a well-characterized route taken by a number of exogenous viruses and toxins following endocytosis (Spooner et al., 2006). Nuclear transport of EGFR depends on Sec61β (Liao and Carpenter, 2007), suggesting that the transmembrane receptor may be removed from the ER membrane via the Sec61 translocon that removes misfolded proteins from the ER lumen for degradation by the proteasome. The EGFR may escape this fate by association with chaperones before importin 1β-dependent nuclear import via the nuclear pore complex (Lo et al., 2006). A pool of Sec61β has also been found on the inner nuclear membrane leading to the suggestion that EGFR may traffic to the inner nuclear membrane via the nuclear pore and then undergo Sec61-dependent removal from the membrane into the nucleoplasm (Wang et al., 2010b).

We previously found that UVC and the chemotherapeutic drug cisplatin induce internalization of EGFR in HeLa cells, and that this internalization can be prevented by an inhibitor of p38 MAPK α and β (also known as MAPK14 and MAPK11, respectively). However, the primary destination of the internalized receptor was perinuclear multivesicular endosomes/bodies (MVBs), rather than the nucleus (Tomas et al., 2015). Nevertheless, we showed that signaling from EGFR in perinuclear MVBs delayed onset of UVC and cisplatin-induced apoptosis. In the current study, we aimed to investigate the relationship between DNA damage, endocytosis and nuclear transport of EGFR. We initially focused on DNA damage induced by X-rays as this has been reported to induce rapid nuclear translocation of a large proportion of cellular EGFR and to be associated with EGFR-dependent promotion of DNA repair.

## RESULTS

### X-ray treatment does not induce significant nuclear translocation of EGFR in HeLa or SCC47 head and neck cancer cells

**Fig. 1.**
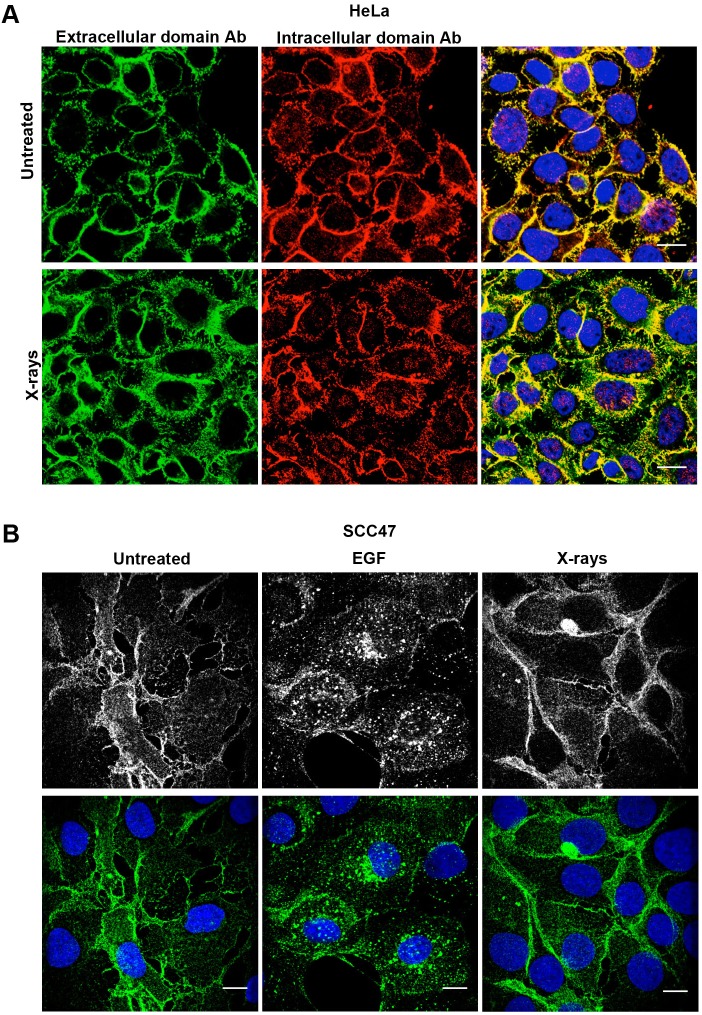
**Immunofluorescence of permeabilized HeLa and SCC47 cells does not reveal increased nuclear EGFR following X-ray treatment.** (A) HeLa cells were serum starved, treated with a single dose of X-rays (4 Gy) and then incubated for 30 min before fixation, permeabilization and staining with anti-EGFR extracellular domain (green) and cytoplasmic domain (red) antibodies, and DAPI (blue). Although a small amount of EGFR staining overlaps with DAPI-stained nuclei this is present in both control and X-ray-treated cells. (B) SCC47 cells were serum starved and left untreated, incubated with EGF for 30 min or were X-ray treated (4 Gy) followed by a 30 min incubation before fixation, permeabilization and staining with anti-EGFR cytoplasmic domain antibody (green) and Hoechst 33342 (blue). EGFR is distributed in puncta throughout the cells after EGF stimulation but remains predominantly associated with the plasma membrane following X-ray treatment. Scale bars: 10 μm.

Immunofluorescence (IF) of HeLa cells using both the extracellular and cytoplasmic domain anti-EGFR antibodies showed that the majority of EGFR staining was localized to the plasma membrane in both untreated controls and cells exposed to 4 Gy X-rays, a typical patient dose (Fig. 1A). A few anti-EGFR-positive puncta overlaid DAPI-labeled nuclei in control cells, but this was not perceptibly increased following X-ray treatment. To determine whether HeLa cells are unusual in their response to X-rays, SCC47 cells, which are a head and neck cancer cell line where the EGFR is a relevant therapeutic target, were also investigated (Fig. 1B). EGF stimulation of these cells led to punctate anti-EGFR staining throughout the cytoplasm after 30 min of EGF treatment, as previously demonstrated in many cell types. In contrast, 30 min after X-ray treatment the majority of EGFR remained localized to the plasma membrane, as in control cells.

**Fig. 2.**
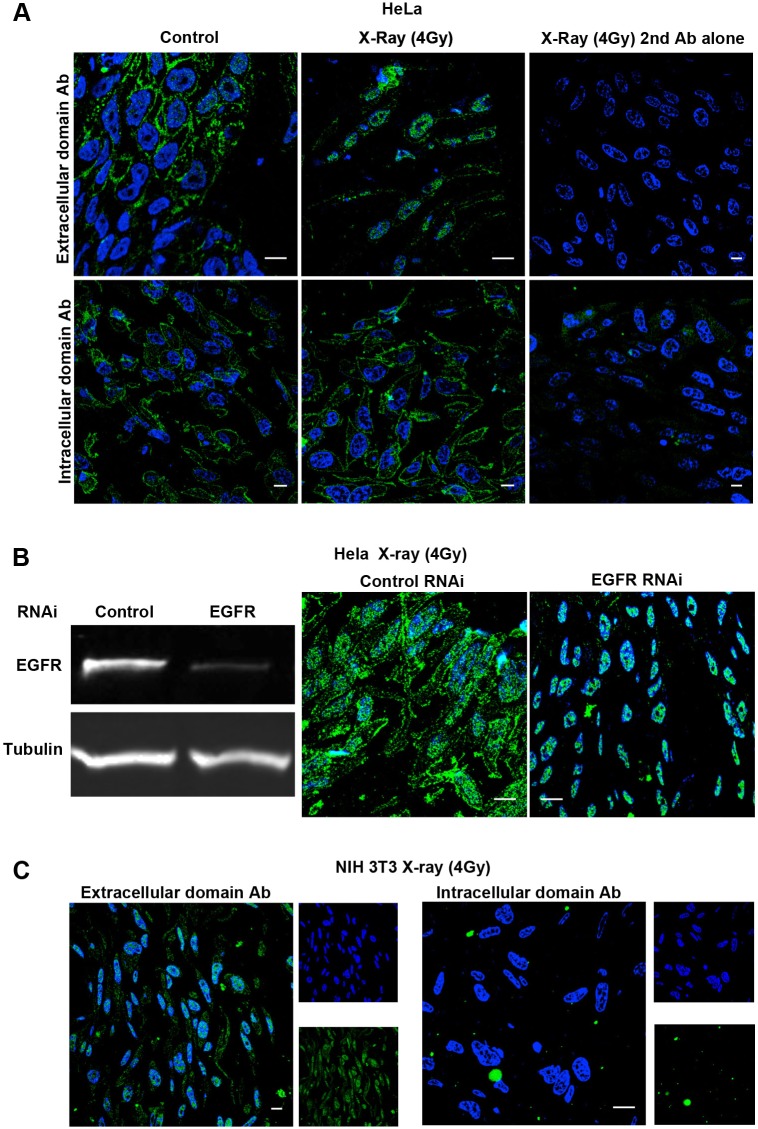
**Apparent increase in nuclear staining of EGFR on sections of X-ray-treated cells.** (A) HeLa cells were serum starved, and treated with or without a single dose of X-rays (4 Gy) followed by a 30 min incubation. Cells were then fixed, cryo-preserved and embedded, and semi-thin cryosections were labeled with anti-EGFR extracellular domain or cytoplasmic domain antibodies (green) and DAPI (blue). Clear nuclear staining is evident with the extracellular domain antibody that is increased following X-ray treatment and absent after labeling with anti-cytoplasmic domain or no primary antibody. (B) HeLa cells treated with control or EGFR-targeting siRNA were western blotted with anti-EGFR antibody to assess efficiency of knockdown or were treated with X-rays (4 Gy) and stained with anti-extracellular domain antibody as in A. Although plasma membrane staining was greatly reduced following EGFR depletion, nuclear staining with anti-extracellular domain antibody was unaffected. (C) NIH 3T3 cells lacking EGFR were treated with X-rays and stained as in A. Nuclear but not plasma membrane staining was clearly present on sections stained with extracellular but not cytoplasmic domain antibody. Scale bars: 10 μm.

A few anti-EGFR-positive puncta did overlay the nucleus in both control and X-ray-treated HeLa cells. Although confocal microscopy allows optical sections to be analyzed, it remains possible for punctate staining that lies immediately below or above the nucleus to appear nuclear, especially as the nucleus is not a perfect sphere and endosomal puncta can lie in nuclear ‘dimples’. Furthermore, the nucleus is known to be difficult to permeabilize effectively, raising the possibility that the scarcity of nuclear staining following X-ray treatment could be due to a problem of antibody accessibility. To overcome these potential issues, we prepared cryosections of HeLa cell pellets using a cryo-ultramicrotome that allows semi-thin (0.5 µm) sections to be cut, which are too thin to contain the full thickness of the nucleus, meaning that signal that overlays DAPI staining must be in the nucleus. Furthermore, immunolabeling of the surface of thawed cryosections with anti-EGFR antibody overcomes the need for the nucleus to be permeabilized. An anti-EGFR antibody against the extracellular domain of the receptor revealed some nuclear staining, which was increased following X-ray treatment but no nuclear staining in either control or X-ray-treated cells was present with a cytoplasmic domain anti-EGFR antibody (Fig. 2A).

To determine whether the nuclear staining obtained with the extracellular domain antibody represented bona fide EGFR staining, HeLa cells were depleted of EGFR using siRNA against the EGFR untranslated region (UTR). Western blotting showed that this treatment removed ∼80% of the cellular EGFR content, and immunofluorescent labeling of semi-thin cryosections revealed a clear loss of plasma membrane staining in X-ray-treated cells (Fig. 2B). However, the nuclear staining was undiminished by siRNA treatment, raising the possibility that the nuclear staining was not derived from the EGFR. siRNA-mediated depletion is not 100% effective and is less effective against long-lived proteins and so the possibility remained that either the nuclear pool for EGFR was a long-lived one that was resistant to siRNA or that the pool of EGFR that remains after RNAi treatment is sufficient to give a nuclear signal. We therefore turned to NIH3T3 cells, which lack EGFR. In these cells, although no plasma membrane or cytoplasmic staining was obtained with either extracellular or cytoplasmic domain antibodies, the extracellular domain antibody gave a strong nuclear signal following staining of ultrathin cryosections of X-ray-treated cells (Fig. 2C). Taken together, these results suggest that the majority of EGFR does not enter the nucleus in HeLa and SCC47 cells after X-ray treatment but some anti-EGFR antibodies can recognize non-EGFR epitopes in the nucleus that become more accessible after X-ray treatment.

**Fig. 3.**
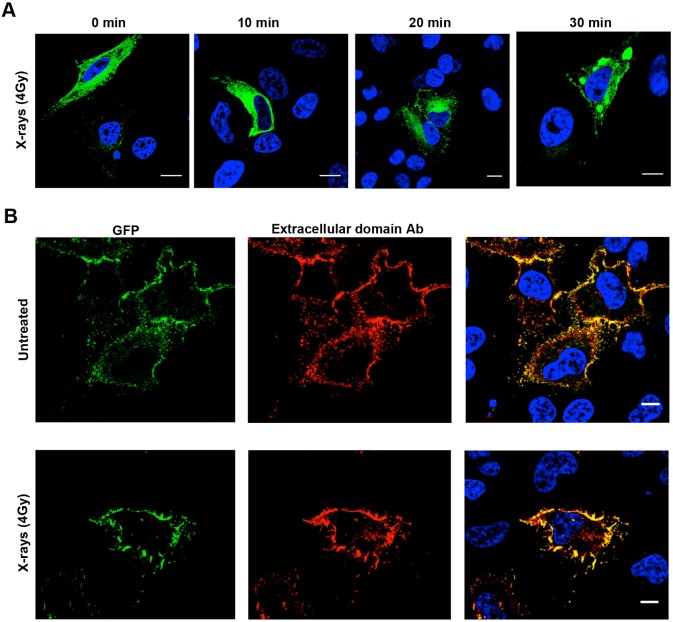
**No evidence of nuclear transport of EGFR–GFP.** HeLa cells were transiently transfected with EGFR–GFP, serum starved, treated with a single dose of X-rays (4 Gy) and chased for the indicated times. (A) GFP fluorescence (green) of highly expressing cells. (B) Cells were fixed, permeabilized and stained for EGFR (red). Highly expressing cells (A) exhibit EGFR–GFP in the biosynthetic pathway as well as the plasma membrane, whereas in cells that expressed less EGFR–GFP (B) it is largely confined to the plasma membrane. No fluorescence was discernible in the nucleus even after X-ray treatment. Scale bars: 10 μm.

As antibody staining is subject to the limitations and variations that arise from the characteristics of the particular antibody used (as exemplified by the experiments described above), we attempted to measure X-ray-induced nuclear trafficking of EGFR using expressed EGFR–GFP, which can be visualized without the use of antibodies. This chimera has been shown to undergo ligand-stimulated endocytosis and signaling indistinguishably from the wild-type protein (Carter and Sorkin, 1998). As shown in Fig. 3A, at very high levels of expression transiently transfected EGFR–GFP can be visualized in the biosynthetic pathway as well as at the plasma membrane but, even at these very high expression levels, EGFR–GFP could not be detected in the nucleus in untreated or X-ray-treated cells. At lower levels of expression, EGFR–GFP remained predominantly plasma membrane-located following X-ray treatment (Fig. 3B).

### Stimuli that induce DNA damage differ in their ability to induce EGFR internalization in HeLa cells

**Fig. 4.**
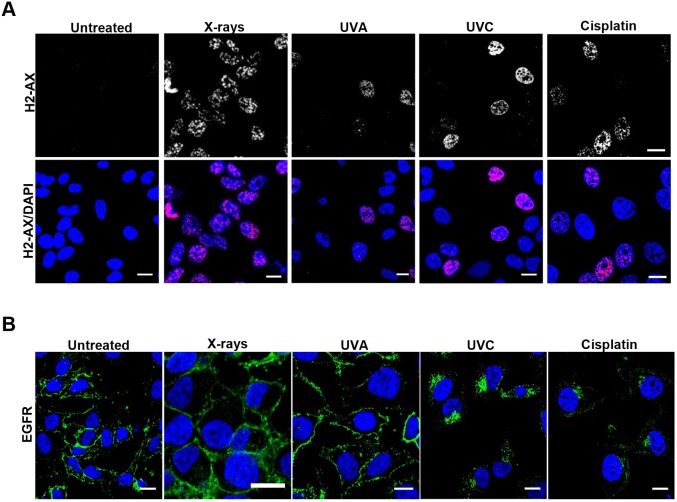
**UVC and cisplatin, but not UVA or X-rays, induce intracellular EGFR accumulation though all induce DNA damage.** (A) HeLa cells were serum starved, and treated with a single dose of X-rays (4 Gy), UVA (10,000 J/m^2^) or UVC (100 J/m^2^) and subsequently incubated for 1 h, or were treated for 6 h continuously with 200 µM cisplatin. Cells were then fixed, permeabilized and stained for γH2AX (H2-AX, red) and with DAPI (blue). (B) HeLa cells were treated as above but were stained for EGFR (green) and DAPI (blue). UVC and cisplatin, but not X-rays or UVA induced endocytosis and accumulation of EGFR in a perinuclear compartment. Scale bars: 10 μm.

**Fig. 5.**
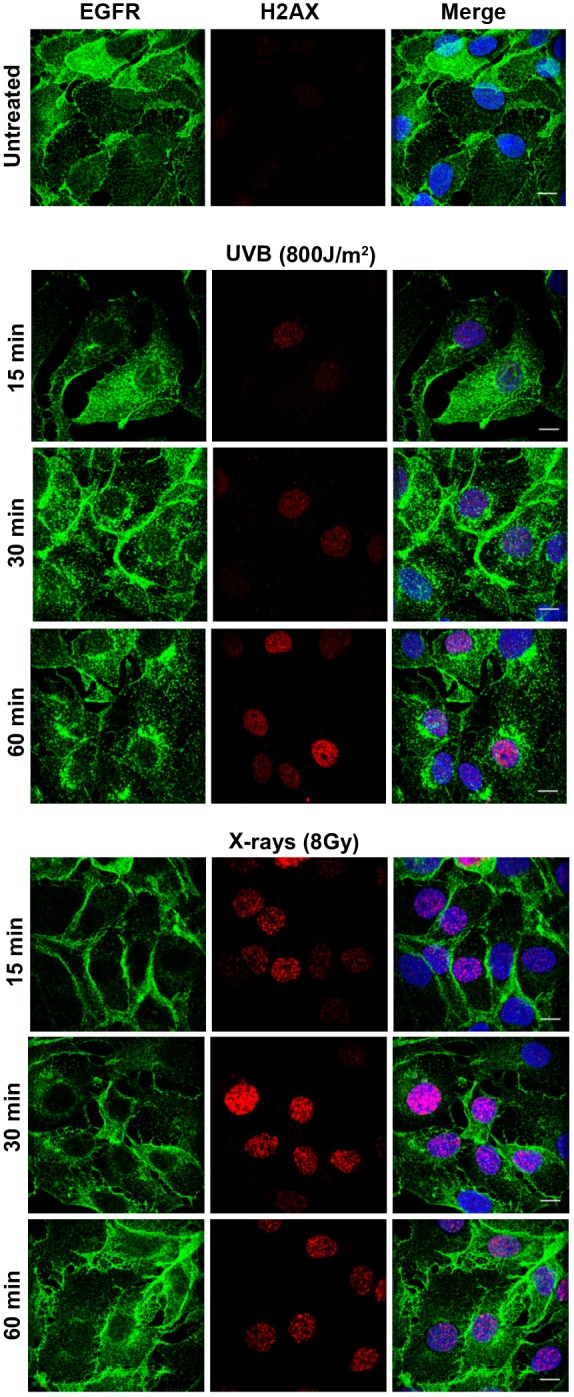
**UVB, but not X-rays, induce substantial intracellular EGFR accumulation in SCC47 cells.** SCC47 cells were serum starved and then were left untreated or treated with a single dose of UVB or X-rays followed by chase for the indicated times. Cells were fixed, permeabilized and stained for EGFR (green) and γH2AX (H2-AX, red) to detect DNA damage. Although both UVB and X-rays induced DNA damage, only UVB induced endocytosis and accumulation of EGFR in a perinuclear compartment. Scale bars: 10 μm.

**Fig. 6.**
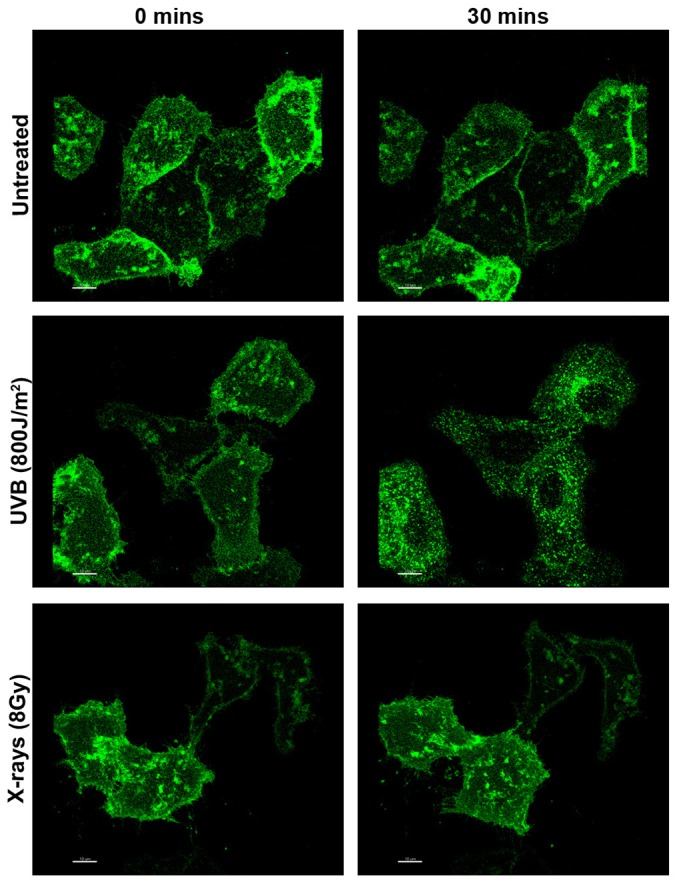
**UVB, but not X-rays, induce endocytosis of EGFR–GFP in HeLa cells.** HeLa cells transiently transfected with EGFR–GFP were left untreated, or treated with UVB (800 J/m^2^) or X-rays (8 Gy). Cells were imaged for 30 min with the first frame taken at 3.5 min and 7.5 min after UVB and X-ray irradiation respectively, and images were taken every 90 s (see Movies 1–3). In untreated and X-ray-treated cells there is little change in EGFR–GFP distribution but EGFR–GFP redistributes from the cell surface to the cell interior after UVB treatment. Scale bars: 10 µm.

The results above indicate that not only did X-rays fail to induce significant translocation of EGFR to the nucleus but that the majority of the EGFR remained on the cell surface. This was surprising as endocytosis and nuclear transport of EGFR has been linked to DNA repair following X-ray (and other) stimuli. In addition, although we did not detect nuclear transport, we had previously been able to detect substantial endocytosis following other stress inducers that cause DNA damage (UVC and cisplatin) (Tomas et al., 2015). To determine whether DNA damage is sufficient to induce EGFR internalization in HeLa cells, the effects of X-rays, cisplatin, UVA and UVC were compared. Staining of phosphorylated histone 2AX (H2AX, also known as H2AFX; the phosphorylated form is denoted γH2AX), which is recruited to sites of DNA damage, demonstrated that all of these stress-inducers caused DNA damage under the conditions used (Fig. 4A). However, immunofluorescent anti-EGFR staining showed that although UVC and cisplatin induced perinuclear accumulation of EGFR, in UVA- and X-ray-treated HeLa cells anti-EGFR staining remained predominantly on the plasma membrane (Fig. 4B). Consistently, quantification of EGFR surface levels through ‘in-cell western’ experiments demonstrated an ∼50% reduction in surface EGFR following UVC but not UVA or X-ray treatment (Fig. S1). Interestingly, although X-ray treatment did not induce redistribution of EGFR from the plasma membrane to endosomes in SCC47 cells, UVB treatment did induce perinuclear EGFR accumulation in these cells while both treatments induced comparable levels of DNA damage (Fig. 5). Redistribution of EGFR from the cell surface to intracellular puncta following cisplatin, UVB and UVC treatment is consistent with the induction of EGFR endocytosis under these conditions. In a previous study, we confirmed the identity of the intracellular compartments in which EGFR accumulated following UVC and cisplatin treatment as multivesicular endosomes/bodies (MVBs) in which anti-EGFR extracellular domain antibody coupled to colloidal gold fed from the cell surface was internalized following these treatments. Here, we also show that cycloheximide treatment to block *de novo* EGFR synthesis does not prevent intracellular EGFR accumulation, indicating that this pool of EGFR is derived from endocytosis, rather than from the biosynthetic pathway (Fig. S2). Finally, to directly demonstrate EGFR endocytosis, we followed the trafficking of EGFR–GFP when expressed in HeLa cells in real-time after UVB and X-ray treatment. EGFR–GFP in untreated (Fig. 6, Movie 1) and X-ray treated (Fig. 6, Movie 2) cells remained largely on the plasma membrane whereas UVB treatment (Fig. 6, Movie 3) induced rapid endocytosis of EGFR into large intracellular puncta.

Thus, in both HeLa and SCC47 cells, doses of UVA and X-rays that induce DNA damage do not induce detectable EGFR internalization whereas UVC, cisplatin and UVB do. Therefore DNA damage per se is not sufficient to induce major EGFR internalization.

### EGFR internalization following treatment with DNA damage-inducing agents correlates with sustained p38 activation in HeLa and SCC47 cells

**Fig. 7.**
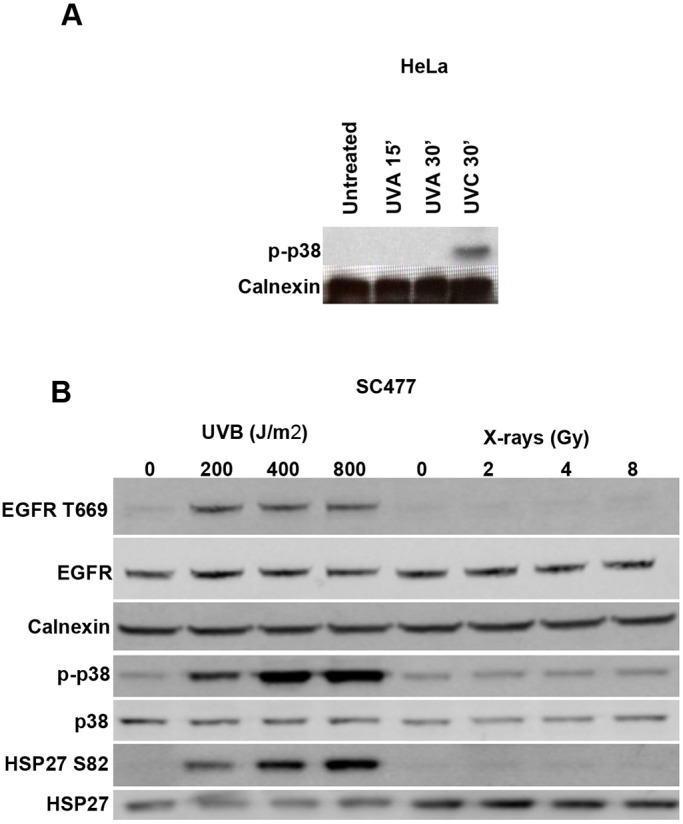
**UVC and UVB, but not X-rays or UVA, induce sustained p38 activation.** (A) HeLa cells were serum starved and treated with a single dose of UVC (100 J/m^2^) or UVA (10,000 J/m^2^), and then chased for 15–30 mins as indicated. Cell lysates were blotted with antibody against phosphorylated p38 (p-p38). Calnexin was used as a loading control. Note that UVC, but not UVA, induces p38 activation. (B) SCC47 cells were serum starved and treated with a single dose of UVB, and then chased for 60 min. Cell lysates were blotted with the indicated antibodies. Note that UVB, but not X-rays, induced p38 activation and phosphorylation of the p38 substrates EGFR T669 and Hsp27.

It was previously shown that UVC or cisplatin treatment of HeLa cells induces substantial activation of p38 proteins, which is required for EGFR endocytosis (Grandal et al., 2012; Vergarajauregui et al., 2006; Zwang and Yarden, 2006). To determine whether treatments that fail to induce detectable EGFR endocytosis also fail to induce p38 activation, the ability of different stimuli to activate p38 proteins was compared. In HeLa cells doses of UVA (Fig. 7A) that induce DNA damage failed to induce detectable p38 activation, as measured by western blotting with an antibody that detects phosphorylated active p38, in contrast to the clear signal obtained in UVC-treated cells. Similarly, in SCC47 cells UVB treatment but not X-rays induced detectable p38 phosphorylation (Fig. 7B). P38 activation following UVB, but not X-ray treatment was confirmed by the demonstration that UVB treatment induced phosphorylation of EGFR T669, and HSP27 S82 (also known asHSPB1), both known p38 phosphorylation sites.

### Does p38 activation and EGFR internalization following stress require ROS?

**Fig. 8.**
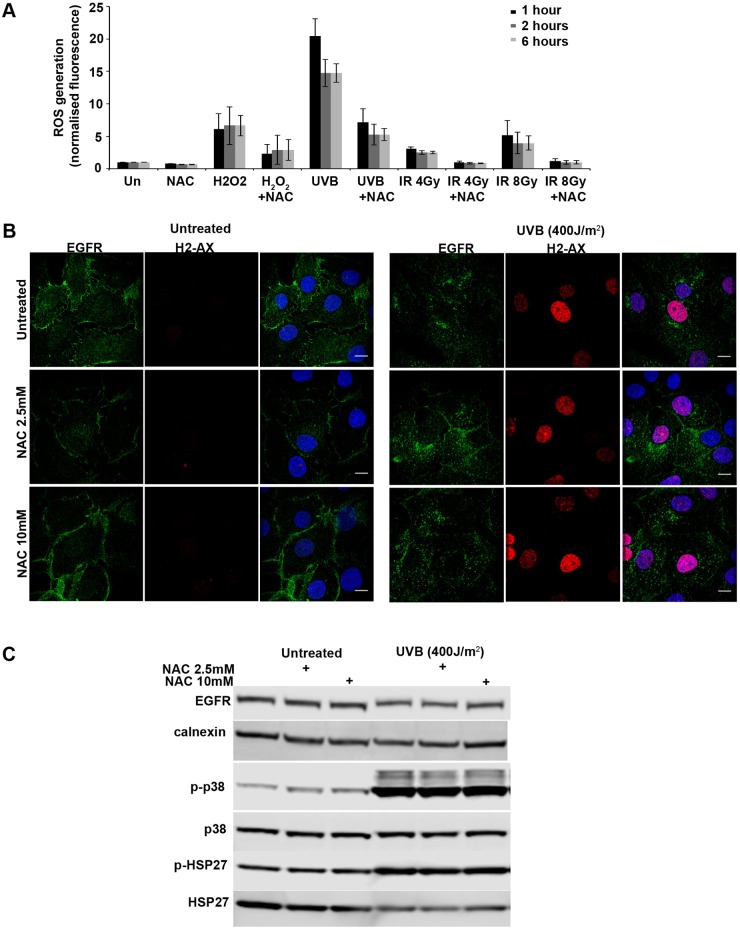
**UVB induces more ROS production than X-rays but ROS scavengers do not prevent EGFR endocytosis.** (A) SCC47 cells were serum starved and treated with DCFDA cellular ROS detection solution for 45 min, then treated with 2.5 mM NAC, 1 mM H_2_O_2_, X-rays and UVB as indicated, and chased for the indicated times. Un, untreated. UVB induced greater and more prolonged levels of cellular ROS than X-rays, which were reduced by incubation with the ROS scavenger NAC. Results are mean±s.e.m. of three observations. (B) SCC47 cells were serum starved, treated with NAC for 60 min, UVB irradiated and chased for 60 min. Cells were then fixed, permeabilized and stained for γH2AX (H2-AX, red) and EGFR. NAC did not prevent endocytosis of EGFR. Scale bars: 10 μm. (C) SCC47 cells were treated with NAC and UVB as in B. Cell lysates were western blotted with the indicated antibodies. NAC did not prevent UVB-induced activation of p38.

p38 can be activated by reactive oxygen species (ROS). X-rays and UV would be expected to induce ROS production, raising the question of why X-rays fail to induce p38 activation. We therefore used the cell permeant reagent 2′,7′-dichlorofluorescin diacetate (DCFDA), a fluorogenic dye that measures hydroxyl, peroxyl and other ROS activity within the cell, to determine whether X-rays and UVB differ in the extent or persistence of the cellular ROS that they induce. As shown in Fig. 8A, although ROS were detectable for at least 6 h after X-ray treatment, UVB induced at least a 5-fold greater level of detectable ROS production. Treatment with the oxygen scavenger N-acetyl-L-cysteine (NAC), reduced UVB-induced cellular ROS levels, such that they were similar to that induced by a high dose of X-rays (8 Gy). We had already shown that this dose of X-rays failed to induce detectable EGFR endocytosis (Fig. 5). However, treatment with NAC did not prevent UVB-induced EGFR endocytosis (Fig. 8B). Furthermore, NAC treatment did not prevent UVB-induced p38 activation, as shown by western blotting with antibodies against phospho-p38 and the phosphorylated p38 substrate Hps27 (Fig. 8C). These results indicate that the ability of UVB but not X-rays to induce p38 activation and consequent EGF endocytosis cannot be explained solely by elevated levels of UVB-induced ROS production.

## DISCUSSION

Many studies have reported nuclear transport of EGFR in cultured tumor cells exposed to ligand (Lin et al., 2001), X-rays (Dittmann et al., 2005, 2008; Liccardi et al., 2011), UVC (Xu et al., 2009) or cisplatin (Liccardi et al., 2011). Although many of these studies have demonstrated increased levels of full-length EGFR in nuclear fractions following treatment with different stimuli, this approach relies on the purity of these nuclear fractions. Therefore, most studies have also shown increased nuclear staining following immunofluorescent or immunohistochemical analysis. The latter is sometimes the only available method, for example when analyzing the distribution of EGFR in tumor specimens, where subcellular fractionation may not be possible. Many factors could explain our inability to clearly detect nuclear EGFR following X-ray treatment. A lack of nuclear staining by immunofluorescence could be due to a failure to effectively permeabilize the nucleus or nuclear ‘soluble’ EGFR could have an altered conformation rendering it refractive to staining with certain antibodies. Interaction partners specific to the nuclear pool of EGFR could mask epitopes, or proteolytic cleavage of EGFR before or after nuclear import may result in loss of epitopes. Finally, small amounts of EGFR in the nucleus that are below the detection limits of our immunofluorescence assays may nevertheless have important nuclear functions. Our study raises, however, an important consideration in the detection of nuclear EGFR, in that some EGFR antibodies detect epitopes in the nucleus that are present after siRNA-mediated EGFR depletion, and in cells that do not express EGFR. In our hands, these epitopes are more accessible when labeling cell sections, rather than whole permeabilized cells, and are increased upon X-ray treatment. This highlights the importance of performing rigorous controls, as in this study, when analyzing the presence or absence of nuclear EGFR. Furthermore, it is noteworthy that nuclear EGFR staining in tumor tissue sections has been reported with some, but not all, anti-EGFR antibodies (Xia et al., 2009), and in this situation it may be more difficult to verify the specificity of staining against EGFR-negative samples. The demonstration of nuclear transport of a fluorescent EGFR chimera, thus overcoming the need for antibody staining, would go a long way towards answering the nuclear import skeptics. However, we were not successful in demonstrating nuclear import with the EGFR–GFP chimera approach. In summary, our study does not exclude the possibility that nuclear transport of EGFR can occur, but highlights the danger of relying on immunostaining as sole indication of it.

Although small amounts of EGFR may endocytose following X-ray treatment in HeLa and SCC47 cells, we found that the majority of EGFR remains on the cell surface. This is in marked contrast to the UVC-induced endocytosis of EGFR that we (Tomas et al., 2015) and others (Grandal et al., 2012; Zwang and Yarden, 2006), have observed. In our previous studies, we used UVC treatment to investigate the pathway followed by EGFR following exposure to stress because UVC-induced endocytosis was well characterized and induces rapid and synchronous endocytosis. We were also able to demonstrate p38-dependent endocytosis following treatment with the chemotherapeutic cisplatin. In the present study, we extended this analysis to include other DNA damage-inducing stressors, which are either encountered during daily life (UVA and UVB) or are commonly used cancer therapies (X-rays and cisplatin). Although all agents induced DNA damage in the majority of cells, UVC, UVB and cisplatin, but not UVA or X-rays, induced detectable EGFR endocytosis and accumulation in a perinuclear compartment. Why do some DNA damage-inducing agents induce EGFR internalization and some do not? Inhibition of p38 was previously shown to inhibit UVC and cisplatin-induced EGFR endocytosis (Vergarajauregui et al., 2006; Winograd-Katz and Levitzki, 2006; Zwang and Yarden, 2006). Here, we show that the ability of DNA damage-inducing agents to cause internalization correlates with p38 activation. We cannot eliminate the possibility that transient p38 activation following X-ray or UVA treatment might induce EGFR endocytosis followed by rapid recycling, as has been previously demonstrated following stimulation with TNFα (Zwang and Yarden, 2006), but we were unable to detect endocytosed EGFR even after short times (<10 min) following X-ray treatment.

Why do some DNA damage-inducing stimuli activate p38 and some do not? Like all MAPK, p38 proteins are activated through phosphorylation by MAPK kinases (MKK3 and MKK6, also known as MAP2K3 and MAP2K6, respectively), which in turn are activated by MAPK kinase kinases (M3Ks) (Son et al., 2013). ASK1 (also known as MAP3K5) is a M3K playing a major role in oxidative stress-induced activation of p38 (Shiizaki et al., 2013). Oxidation of the ASK1-associated protein thioredoxin, releases it from ASK1 allowing ASK1 oligomerization, autophosphorylation and activation. Although X-rays are known to induce ROS production, the extent to which they do so under the conditions used in this study is much less than UVB, suggesting that a threshold amount or duration of ROS production is required to activate p38 M3Ks. However, although incubation with a ROS scavenger reduced UVB-induced cellular ROS, it did not prevent UVB-induced endocytosis or p38 activation, indicating that additional factors regulate p38 activation following UV treatment. p38 activity is also regulated through inactivation by a number of phosphatases (Son et al., 2013). Differential regulation of an inactivating phosphatase might also contribute to the inability of X-rays and UVA to induce sustained p38 activation.

How does sustained p38 activation promote EGFR endocytosis? We previously showed that p38 activity is required not only for promoting clathrin/AP2-dependent endocytosis from the plasma membrane but is also required to retain the EGFR in perinuclear MVBs (Tomas et al., 2015). p38-mediated EGFR T669 phosphorylation may induce a conformational change in the EGFR cytoplasmic domain, revealing the AP2 interaction sites that are necessary for p38-dependent endocytosis. We previously showed that, although the UVC-mediated activation of p38 and retention in perinuclear MVBs is sustained for many hours, the p38-mediated phosphorylation of EGFR at T669 is not. It is likely, therefore that p38-mediated phosphorylation of substrates in addition to the receptor itself regulate EGFR trafficking. Notably, a recent paper on opioid receptor recycling showed that the Rab5 effectors, EEA1 and rabenosyn 5, are targets of p38 and phospho-mimetic mutants of EEA1 can bypass the requirement for p38 in opioid receptor endocytosis (Mace et al., 2005).

Our demonstration that EGFR endocytosis is not a response to DNA damage, and does not necessarily result in nuclear accumulation, suggests that EGFR-stimulated DNA repair may not depend on endocytosis or nuclear import. Nuclear accumulation could facilitate interaction with DNA-PK. However, the presence of a cytoplasmic pool of DNA-PK potentially renders this substrate of the EGFR kinase accessible without EGFR endocytosis and nuclear translocation. Alternatively, very low levels of EGFR endocytosis and nuclear transport may be sufficient to activate DNA repair. If not linked with DNA damage/repair, what is the function of the endocytosis and perinuclear accumulation of a major part of the cellular complement of EGFR? We previously showed that internalization of EGFR following cisplatin or UVC treatment was necessary to induce a low level of sustained EGFR activation and downstream signaling, which delayed the onset of apoptosis (Tomas et al., 2015). We did not, however, determine the downstream signaling pathways that were necessary for delayed apoptosis. This is an important topic for future study.

We have shown that sustained p38 activation, but not DNA damage, is sufficient to induce EGFR endocytosis and perinuclear accumulation of EGFR. Although intracellular EGFR accumulation delays the onset of apoptosis, it is unable to prevent cell death, which may in fact be induced by the prolonged p38 activation. Thus, although perinuclear accumulation of EGFR can bring about short-term protection, in practice, it may be that perinuclear accumulation of EGFR in cells in a tumor indicates a state of prolonged p38 activation that precedes cell death. On the other hand, nuclear EGFR has been implicated in promotion of proliferation and DNA repair and has been associated with poor prognosis, suggesting that nuclear EGFR may indicate a different cellular outcome to that suggested by perinuclear EGFR. This highlights the importance of understanding how trafficking of EGFR regulates EGFR signaling and the extent to which the subcellular distribution of EGFR predicts tumor cell fate. High resolution and carefully controlled analysis of the subcellular distribution of EGFR in tumors and analysis of the effect of DNA damage-inducing therapies on that distribution will shed light on the role of EGFR trafficking in regulating and predicting therapeutic outcomes.

## MATERIALS AND METHODS

### Reagents and antibodies

Cisplatin (200 μM) was from Mayne Pharma. Anti-EGFR antibodies used for immunofluorescence were against the extracellular domain antibody purified from the mouse 108 hybridoma (ATCC; 1:200), and rabbit anti-cytoplasmic domain antibodies (4267 Cell Signaling; 1:50). Antibodies used for western blotting were: anti-EGFR #2239 (1:1000), anti-phospho-EGFR-Y1068 #2234 (1:500), anti-phospho-EGFR-T669 #3056 (1:500), anti-p38 #9217 (1:2000), anti-phospho-p38 #4511 (1:1000), anti-Hsp27 #2402 (1:1000), anti-phospho-Hsp27 #9709, and anti-calnexin #2433 (1:1000) all from Cell Signaling. Anti-γH2AX #05-636 (1:100) for immunofluorescence was from Millipore.

### Cell culture, treatments and transfections

HeLa cells were from ATCC, NIH3T3 cells were from CR-UK London Research Institute and UM-SCC47 head and neck cancer cells were generously provided by Dr Tim Fenton (UCL Cancer Institute, London, UK). All cells were cultured in Dulbecco's modiﬁed Eagle's medium (DMEM) in 10% fetal calf serum in 5% CO_2_. Prior to treatment, cells were serum-starved overnight (HeLa and 3T3) or for 2 h (SCC47). For X-ray treatment, cells were exposed to the indicated doses with the A.G.O. HS 321 kV X-ray system at 2 Gy/min and then incubated for the indicated times in serum-free medium. For UV treatment, cells were exposed to 100 J/m^2^, 10,000 J/m^2^, and 800 J/m^2^ of UVC (254 nm), UVA (364 nm) or UVB (312 nm), unless otherwise indicated, with CL-1000 UV crosslinkers (Spectronics corporation) at room temperature immediately after aspirating the medium, and were then incubated for the indicated times in serum-free medium. These treatments took from a few seconds to 3 min, depending upon the crosslinker capacity. For cisplatin treatment, cells were treated continuously with 200 µm cisplatin in serum-free culture medium.

HeLa cells were transiently transfected with EGFR–GFP (Carter and Sorkin, 1998) with Lipofectamine 2000 or 3000 reagent (Life Technologies) following the manufacturer's guidelines, for 48 h. To deplete EGFR, HeLa cells were transfected with siRNA targeting the EGFR UTR (Hs_EGFR_6, QIAGEN) with Lipofectamine RNAiMAX (Life Technologies) for 72 h.

### Immunofluorescence of permeabilized cells

All cells were fixed with 4% paraformaldehyde (PFA). HeLa and NIH3T3 cells were permeabilized with 0.5% Triton X-100 for 10 min, and SCC47 cells with ice-cold 0.2% Triton X-100 for 3 min. For HeLa and NIH3T3 cells, blocking and antibody incubations were in PBS with 1% bovine serum albumin (BSA). SCC47 cells were blocked in 5% BSA with 10% FBS. After labeling with primary antibodies for 1 h at room temperature or overnight at 4°C, cells were incubated with Alexa Fluor-conjugated secondary antibodies for 45–60 min at room temperature. Coverslips were mounted in Prolong Gold antifade reagent with 4,6-diamidino-2-phenylindole (DAPI; Life Technologies) or were incubated with Hoechst 33342 for 30 min at room temperature before mounting in Prolong Diamond (Life Technologies), and images were acquired with a Leica TCS SP2 confocal system with a 63× 1.4 NA oil-immersion objective or a Leica TCS SP3 confocal with a 63× 1.3 NA oil-immersion objective.

### Immunofluorescence of cryosections

Cells were fixed in 4% PFA, quenched with 15 mM glycine, scraped from the dish and cell pellets were embedded in 12% gelatin. Small blocks infused with 2.3 M sucrose at 4°C were mounted on pins and frozen in liquid nitrogen. 0.5 µm sections were cut with a cryo-ultramicrotome at −80°C, and retrieved in sucrose onto glass slides. Sections were blocked and labeled with primary and secondary antibodies and visualized as described above.

### Western blotting

HeLa cells were lysed in lysis buffer [20 mM Tris-HCl, 150 mM NaCl, 1 mM EDTA, 1% NP40 (or Triton-X-100), pH 7.4 plus protease inhibitor cocktail (Calbiochem set I) and phosphatase inhibitor cocktail (Calbiochem set II)]; lysates were fractionated by SDS-PAGE on 10% gels under reducing conditions and immunoblotted onto nitrocellulose membranes. Bands were detected by using enhanced chemiluminescence (Pierce), and exposed films developed on a SRX-101A Film Processor (Konica).

SCC47 cells were lysed with CellLytic M (Sigma-Aldrich) with complete Mini Protease Inhibitor Cocktail tablets (Sigma-Aldrich) and phosphatase inhibitors PhosSTOP (Sigma-Aldrich). Lysates were fractionated by SDS-PAGE on NuPage Novex 4–12% Bis-Tris protein gels (Thermo Fisher) and immunoblotted onto Immobilon-FL PVDF membrane (Millipore). Proteins were detected using the Odyssey Imaging System (Li-Cor).

### Surface down-regulation ‘in-cell western’ assay

Approximately 65,000 cells were seeded with six replicates per condition in a 48-well plate. Cells were serum starved overnight and, following the appropriate treatment, fixed in 4% PFA in PBS for 20 min. Half of the wells were permeabilized in 0.1% Triton X-100 for 8 min to quantify total EGFR level while the other half were left untreated to quantify surface EGFR levels. Cells were processed for in-cell western with the 108 anti-EGFR antibody followed by IRDye 800CW donkey anti-mouse-IgG secondary antibody (LI-COR) and DRAQ5 (to quantify cell number). Images were taken at 700 and 800 nm with a LI-COR Odyssey Infrared Imaging System and processed in ImageJ. Integrated density for the same area was quantified for each well and normalized by cell number.

### Live-cell imaging of EGFR–GFP

HeLa cells 24 h after transfection with EGFR–GFP were serum-starved overnight. The medium was then changed to Cell Imaging Medium [Hank's balanced salt solution without Phenol Red and sodium bicarbonate (Sigma) with 10 mM HEPES (Fisher Scientific), pH 7.4, sterile-filtered] and the cells were irradiated with X-rays or UVB. The images were acquired using a Zeiss LSM880 inverted confocal microscope with a Plan Apochromat 63×1.4 NA oil-immersion objective and a photomultiplier tube. The cells were imaged at 37°C for 30 min starting at 7 min post-X-ray and 3.5 min post-UVB irradiation. Images were acquired every 90 s with a *z*-stack of 3.2 µm-3.6 µm and an interval of 0.4 µm. The 3D movies were prepared using Imaris 8.4 software.

### Detection of ROS

SCC47 cells in 96-well black clear-bottom plates were serum-starved overnight in medium without Phenol Red, then washed with 1× DCFDA buffer [DCFDA cellular ROS detection assay kit (Abcam)] and incubated with DCFDA for 45 min at 37°C in the dark. The cells were washed in PBS and placed in starvation medium without Phenol Red and treated with NAC (2.5 mM,) and H_2_O_2_ (1 mM), then irradiated with UVB or X-rays and incubated for the indicated times. The fluorescence (excitation 485 nm, emission 535 nm) was read with a Varioskan Lux plate reader (Thermo Scientific).
